# A phenomenological study on East and Southeast Asian dental educators: perceived importance, challenges, and strategies in teaching dental materials science

**DOI:** 10.1186/s12903-023-03293-4

**Published:** 2023-08-13

**Authors:** Galvin Sim Siang Lin, Wen Wu Tan, Chan Choong Foong

**Affiliations:** 1 Department of Dental Materials, Faculty of Dentistry, Asian Institute of Medicine, Science and Technology (AIMST) University, Bedong, Kedah 08100 Malaysia; 2 Department of Dental Public Health, Faculty of Dentistry, Asian Institute of Medicine, Science and Technology (AIMST) University, Bedong, Kedah 08100 Malaysia; 3 Medical Education and Research Development Unit (MERDU), Faculty of Medicine, University of Malaya, Kuala Lumpur, 50603 Malaysia

**Keywords:** Dental education, Qualitative research, Dental educators, Dental materials, Thematic analysis

## Abstract

**Background:**

Effective teaching of dental materials science is crucial for dental students to develop a comprehensive understanding of materials used in clinical practice. However, literature on educators’ views on teaching this subject is still scarce. This qualitative study aimed to explore the lived experiences of dental educators in teaching dental materials science subjects, thereby addressing potential gaps and enhancing teaching practices.

**Methods:**

Thirteen dental educators from East and Southeast Asian countries (Malaysia, China, Indonesia, Thailand, South Korea, and Japan) participated in the present study. The present study adopted a transcendental phenomenological approach. One-to-one semi-structured online interviews were conducted. Interviews were recorded and transcribed verbatim. Thematic analysis was employed to identify patterns in the educators’ experiences.

**Results:**

Three themes emerged from the present study. First, perceptions of the importance of dental materials science, highlighting its relevance in clinical practice, patient care, and lifelong learning. Second, the challenges faced in teaching dental materials science include limited instructional time, complex content, and insufficient resources. Third, specific strategies, such as applying interactive teaching methods, integrating clinical scenarios, and promoting critical thinking skills have been suggested to enhance teaching and learning.

**Conclusion:**

Understanding dental educators’ experiences can improve dental materials science education, curriculum development, teaching methods, and faculty training programmes, ultimately enhancing the knowledge and skills of dental students in this field.

**Supplementary Information:**

The online version contains supplementary material available at 10.1186/s12903-023-03293-4.

## Background

Dental materials science plays a crucial role as an important preclinical subject in most dental curricula [1]. It provides students with the knowledge and skills necessary to select and utilise appropriate dental materials for various clinical procedures, thereby ensuring optimal patient care and treatment outcomes [2]. Dental materials, including restorative materials, impression materials, prosthetic materials, and biomaterials, play a fundamental role in various dental procedures such as restorations, prosthodontics, endodontics, and orthodontics. Therefore, dental students must acquire a comprehensive understanding of the physical, mechanical, chemical, and biological properties of commonly used dental materials [3]. They must be familiar with the principles of material selection, manipulation techniques, and the factors influencing material performance and longevity [4]. Moreover, they must develop critical thinking skills to evaluate and apply research findings and advancements in dental materials science in clinical practice [5].

Undeniably, teaching dental materials science presents unique challenges in dental education. The subject matter is multidisciplinary, drawing from fields such as materials science, chemistry, physics, and engineering [6]. It is also one of the preclinical dental subjects that most students regard as bored, monotonous, and difficult [7, 8]. As a result, dental educators must ensure that students grasp the fundamental concepts while relating them to clinical scenarios and real-world applications [9, 10]. Furthermore, dental materials science is a rapidly evolving field with constant advancements in materials technology and research [11]. Thus, dental educators must remain updated with the latest developments and effectively communicate these advancements to the students. Effective education in dental materials science requires a combination of factual knowledge delivery, laboratory exercises, and clinical applications [12]. Lectures provide a theoretical foundation and conceptual understanding, while laboratory sessions allow students to gain practical experience in handling and manipulating dental materials [9]. Clinical integration is essential for bridging the gap between classroom knowledge and its application in patient care [10].

Although dental materials science education is of utmost importance, several challenges exist in its content delivery, such as limited instructional time within crowded curricula, complexity of the subject matter, and availability of resources and laboratory facilities [7, 13]. In addition, the diversity in learning styles and backgrounds among dental students necessitates the use of innovative teaching methodologies and approaches [7, 14]. Effective teaching of this subject is essential for dental students to become competent professionals, and dental educators play a vital role in shaping students’ understanding of dental materials science. Therefore, it is important to investigate dental educators’ experiences of teaching this subject.

Nevertheless, there is a paucity of research exploring the experiences of dental educators in teaching dental materials science. Understanding their perspectives, challenges, and strategies can help to improve dental education by addressing potential gaps and enhancing teaching practices [15]. Hence, the present qualitative study aimed to explore the experiences of dental educators in teaching dental materials science. Valuable insights can be gained to improve dental materials science education, such as enhancing the existing curriculum, and teaching methods, and promoting faculty training programmes, ultimately improving the knowledge and skills of dental students in the field of dental materials science.

## Methods

### Ethical considerations

Ethical approval was obtained from the first author (GSSL) institutional review board (ethical approval code: AUHEC/FOD/2022/23/11/05) prior to data collection. Participants were provided with informed consent forms and assured of the confidentiality and anonymity of their responses.

### Research design

The reporting for the present study adhered to the Consolidated Criteria for Reporting Qualitative Research (COREQ) recommendations [16] (Appendix 1). The research was designed by GSSL and the second author (WWT). Both full-time male faculty lecturers had prior experiences in conducting qualitative research. A qualitative research design was employed to gather rich and in-depth data on dental educators’ experiences. One-on-one semi-structured interviews were conducted to explore their experiences and perspectives. Interviews were performed by the GSSL, who earned a basic dental degree with a postgraduate master’s degree in medical education and a Doctor of Philosophy in Dentistry. The transcendental phenomenology approach according to Edmund Husserl [17] was used to learn and explore dental educators’ experiences in teaching dental materials science. This approach involves examining the lived experiences of participants so that new meanings and appreciations that emerge from their experiences can be developed to inform, or even reorient, and allow outsiders to understand their experiences [18].

### Participants selection

Thirteen dental educators from different dental schools across East and Southeast Asian countries participated in this study. The maximum-variation sampling method was applied. The participants had diverse backgrounds, in terms of their years of teaching experience and educational qualifications. Participants were invited to participate via email or telephone calls. If there was no response after one week, a follow-up email was sent. To avoid bias, the authors did not have any direct relationships with the participants. All the participants agreed to participate in the study. They were informed of the data collection, background, and purpose of the study before consenting to participate in the interviews.

### Data collection

The interviews were conducted using the online Zoom platform, audio-recorded with the participants’ consent, and transcribed verbatim for analysis. The interview guide was pilot tested and included open-ended questions focusing on the participants’ experiences in teaching dental materials science. The interview questions were related to how the participants perceived the importance of dental materials science in current dental education, what challenges have been faced in delivering the content of dental materials science, and strategies for improving the teaching of dental materials science. Each interview session lasted between 20 and 30 min without the presence of a third party. No repeat interviews were conducted and no incentives for participation were provided. The interview data were anonymised and transcribed verbatim by GSSL and WWT. Some interview data contained Mandarin and Malay languages, which were translated into English by the authors prior to the data analysis. The authors are well-versed in Mandarin and Malay languages, and translators were invited to translate and verify the translation. Member-check was also performed. Interview transcripts were sent to participants who agreed to provide feedback for their comments, and no corrections were required.

### Data analysis

Thematic analysis was employed to identify the common themes and patterns in the data. The analysis involved several stages, including familiarisation with the data, coding, theme development, and refinement. During the initial stages of the analysis, GSSL and WWT independently engaged in open coding, where interview transcripts were examined line-by-line using NVivo software (QSR International’s NVivo 10). Ideas that emerged from the data were identified and labelled using descriptive codes. Axial coding was then performed, whereby the authors further analysed the coded data to explore the relationships between the ideas. These ideas were consolidated into themes with the support of interview quotes. This process allowed for a more refined understanding of the dental educators’ experiences. The authors then discussed and finalised the themes and subthemes. Discrepancies were resolved by consensus. Data saturation was reached after interviewing the 11th participants, where no new ideas emerged. However, the interviews continued until the 13th participant, to ensure that all ideas were adequately captured. Representative pseudonymised quotes are used to ensure privacy [19]. Pseudonymisation number P represents participants. Themes and subthemes are illustrated in the form of an explicatory model in a Venn diagram [20].

## Results

The age of the participants ranged from 30 to 65 years. Five participants had less than 5 years of professional experience in teaching dental materials science, six had 6 to 15 years of teaching experience, and the remaining two participants had more than 15 years of teaching experience. Four participants were from Malaysia, three from China, two from Indonesia, two from Thailand, one from South Korea, and one from Japan. All participants were lecturers (or educators) affiliated with their respective dental schools at the time the study was conducted.

**Fig. 1:**
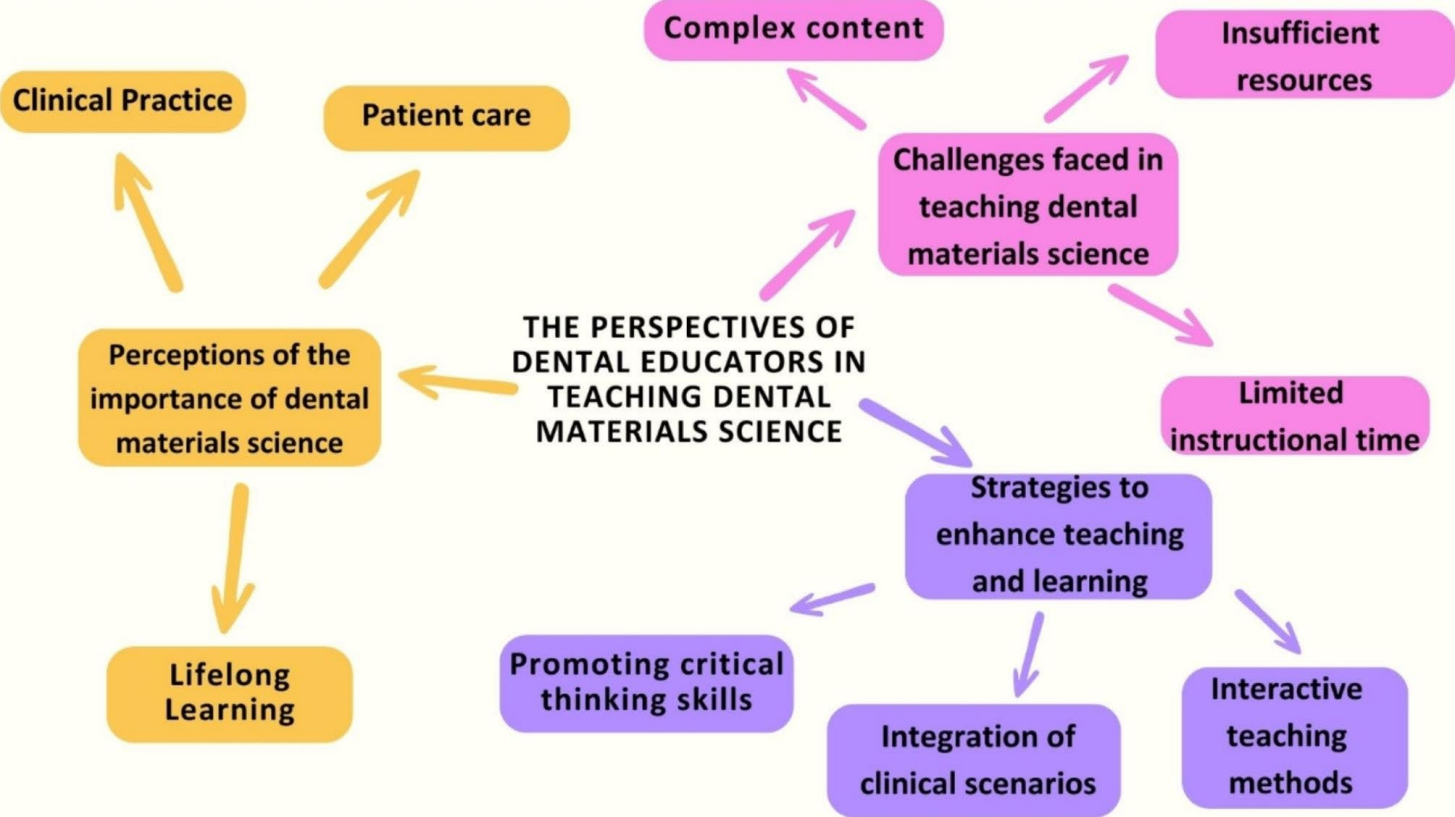
Venn diagram showing the three themes and their subthemes in describing dental educators’ experiences in teaching dental materials science

Three themes emerged from the data: (1). Perceptions of the importance of dental materials science, (2). Challenges faced in teaching dental materials science, and (3). Strategies used to enhance teaching and learning in dental materials science (Fig. 1).

### Theme 1: perceptions of the importance of dental materials science

This theme explored dental materials science educators’ views on the significance of dental materials science in dental education. These sub-themes highlighted the relevance of materials science in clinical practice, patient care, and lifelong learning.

#### Sub-theme 1: clinical practice

Participants felt that dental material science was important for dental students. Dental materials science has contributed to the fundamental concepts in the clinical years. When these students enter clinical years, they would need to use the knowledge to mix and manipulate dental materials, relate the knowledge with clinical practice, and apply the knowledge in their clinical decision-making. Representative quotes are listed below:

P2: *“It is important for students to know how to mix and manipulate dental materials, as they need to use them in their clinical years later.”*

P6: *“I find it very important for this (dental materials science) to be adequately taught; students can utilise their knowledge in clinical practice.”*

P10: *“Dental students must learn dental materials science before they can apply it in the clinical settings.”*

P11: *“Without knowing what the materials (dental materials), they (students) could not apply correctly in the clinical aspects… they should know the fundamental concepts and relate them to their clinical decision-making.”*

#### Sub-theme 2: patient care

Participants believed that learning dental materials science could help dental students improve their patient treatment outcomes by selecting appropriate materials for their patients. If students were able to master dental materials science, they could ensure patient safety and quality of care. Representative quotes are listed below:

P1: *“They (Dental students) can improve patient treatment outcomes if they have the knowledge to select the appropriate dental materials for patients.”*

P4: *“Patient care will definitely be improved if dental students or dental practitioners can use their knowledge in dental materials science wisely.”*

P5: *“I think that patient care is important…so students must know what materials suit them (patients) the best.”*

P7: *“I told my students to do no harm to patients by choosing suitable dental materials.”*

P8: *“If they (students) cannot perform well in the dental materials subject, how can they (students) ensure patient safety and quality of care?”*

#### Sub-theme 3: lifelong learning

Participants commented that dental materials science continues to evolve, and that more advanced materials will be introduced. Thus, they felt that dental students must practice evidence-based dentistry to equip themselves with lifelong learning attitudes. They hoped that students would continue learning new materials after graduation and update themselves with evidence-based knowledge of new materials as dental materials science is evolving. Representative quotes are listed below:

P1: *“Dental materials science is a huge area to cover and continue to evolve… students need to grasp it well and continue to learn advanced materials.”*

P2: *“It is important for students to continue learning the subject as new materials will be marketed every day.”*

P6: *“I told them (students) to continue learning the subject even after they graduated because learning dental materials has no end...”*

P9: *“Students must continue to update themselves with evidence-based knowledge of new materials in order for them to perform well once they finish their study.”*

P10: *“In our country, dental materials are very important, and we held many seminars and conferences to educate students and dentists to improve their knowledge of dental materials.”*

P11: *“We must allow our students to have this lifelong learning attitude towards dental materials science because it is the bread and butter of most dental specialities.”*

P13: *“Nowadays, we are into evidence-based dentistry, so they (students) must equip themselves with the current evidence of the materials applications and continue to learn new materials.”*

### Theme 2: challenges faced in teaching dental materials science

This theme identified the challenges encountered by dental educators in teaching dental materials science. The subthemes included limited instructional time, complex content, and insufficient resources.

#### Sub-theme 1: limited instructional time

Participants found that the time allocated to teaching all content in dental materials science was limited, and it was perceived as minimal and insufficient. Despite the allocation of one or two semesters, they struggled to deliver the content within a short period. They wished for more time to be allocated. Representative quotes are listed below:

P1: *“The main issue is limited teaching time.”*

P2: *“I found that the time allocated for teaching dental materials is minimal.”*

P3: *“There are many things to be covered in just two semesters… absolutely not enough (time).”*

P5: *“Our faculty allocated only one semester for this subject; how can I teach everything?”*

P8: *“The time (available) for us to teach is not enough.”*

P12: *“If we can have more slots allocated to this subject, it would be better to teach.”*

P13: *“Time is a factor that causes most of us to struggle to deliver the content.”*

#### Sub-theme 2: complex content

Participants felt that it was challenging to deliver dental material science content due to its complexity. This subject comprises elements from the fields of chemistry, biology, physics, and biotechnology. Sometimes, students were confused about the sciences behind dental materials. Students provided feedback that the subject was difficult to understand, and some educators felt the same. Participants also found it difficult to understand and explain the subject in a clinical setting.

P2: *“This is challenging because there are many chemistry, biology and physics components in it (dental materials).”*

P4: *“Dental materials, although sounds “dentistry”, the fact that the content primarily includes chemistry and biotechnology is a huge challenge for us (educators) to teach.”*

P6: *“Sometimes, not only are the students confused with the sciences behind these materials, we (educators) also find it difficult to understand.”*

P7: *“My students always tell me that it is a very hard subject, and I will reply to them that I feel the same; some parts are not easy to explain in a clinical setting.”*

P10: *“It is a complex subject…I personally find it difficult to understand the content and challenging for me to deliver and teach my students, don’t you feel the same?”*

#### Sub-theme 3: insufficient resources

The participants mentioned several challenges in terms of resources. First, in some schools, no practical sessions were provided for educators to guide students after they had attended lectures. In some schools, laboratories were not well equipped with machines and materials for experiments. As a result, actual clinical scenarios cannot be shown to students. Second, learning resources, such as dental laboratories, reading materials, and available biomaterials, were lacking in their respective faculties. They also claimed that there was a lack of teaching staff with professional dental and postgraduate qualifications in dental materials science. Educators with these qualifications may be able to help students relate material science to dentistry.

P3: *“We (educators) were only given slots for lectures on teaching dental materials, and there were no practical sessions for us to guide our students.”*

P4: *“Well, the reading materials and learning resources are quite lacking in this field, even the dental conferences that I joined did not cover advanced materials aspects.”*

P7: *“In addition to the students’ learning resources, human resources are also a problem…we could not find dental lecturers with a dental materials background...”*

P8: *“Laboratory facilities in our faculty are not well equipped with all the material testing machines, so it is difficult to show it to the students…”*

P9: *“Our faculty has to hire non-dental experts to teach dental materials because we (faculty) cannot find staffs with a dental degree and postgraduate qualifications in dental materials…so it is challenging for them to teach (dental materials) while relating to dentistry”*

P11: *“We (faculty) do not have sufficient learning tools, such as advanced dental laboratories, biomaterials, and reading materials, for our students to learn more in dental materials science.”*

### Theme 3: strategies to enhance teaching and learning in dental materials science

This theme discussed strategies suggested by dental educators to enhance teaching and learning in dental materials science. The sub-themes encompassed the use of interactive teaching methods, integration of clinical scenarios, and promotion of critical thinking skills.

#### Sub-theme 1: interactive teaching methods

Participants agreed that lectures should not be the only teaching method and that students’ active engagement is desirable. They suggested making teaching and learning sessions more interactive by adding more practical sessions, incorporating artificial intelligence, team-based learning, and case-based learning, or providing feedback to students rather than lectures alone. Subsequently, students would not feel bored when learning about dental materials science.

P1: *“I hope that the teaching and learning sessions can be more interactive…not just lectures alone.”*

P2: *“Maybe we can add more practical sessions for them to learn how to mix the materials.”*

P3: *“I think we can add more AI (artificial intelligence) in our (educators) teaching.”*

P6: *“I have seen an article using team-based and case-based learning, which maybe can try that out.”*

P9: *“Our faculty encourages us (educators) to find new teaching methods.”*

P10: *“I would recommend adding feedback to teaching dental materials.”*

P12: *“If we have different teaching styles, students may not feel bored.”*

P13: *“I am against using lecture as a teaching tool, we need (to have) more active engagement with the students.”*

#### Sub-theme 2: integration of clinical scenarios

Participants commented that it was the right time to change their school of thought. They suggested that the dental curriculum should include both preclinical and clinical aspects in the teaching of dental materials science. Thus, it will close the gap between learning (preclinical) knowledge and clinical application. Specifically, clinical scenarios can be integrated into preclinical studies. As a result, students would be able to use (preclinical) knowledge to solve clinical cases. Participants foreseen that their students would be happy to see these changes in the curriculum.

P2: *“I also think that if we (educators) can add some clinical discussions, that will be better.”*

P4: *“Break down the curriculum and teach dental materials across preclinical and clinical years to allow students to use their knowledge in clinical cases.”*

P6: *“It is time to change the old school of thought and incorporate more clinical teaching for this subject (dental materials).”*

P7: *“My students would be very happy if they had more clinical aspects in learning this (dental materials).”*

P10: *“Using case-based learning…I think this can close the gap between preclinical and clinical teaching in dental materials science.”*

#### Sub-theme 3: promoting critical thinking skills

Participants believed that the teaching of dental materials science should promote critical thinking among students, and that it is their responsibility to guide them in developing critical thinking. Consequently, educators can conduct group discussions, problem-based learning, and case studies among their students. For the participants, students must not memorise textbooks; instead, they must be able to justify the selection of dental materials.

P3: *“Afterall, they (students) need to think critically about which materials to use, and we need to guide them (students) on how to have such skills.”*

P5: *“...can think critically by allowing them to have more group discussions.”*

P7: *“They not only need to know how to mix, but also how to justify the selection of each material critically with what they have learned.”*

P8: *“It is important to change the teaching method so that we (educators) can help them (students) achieve a higher level in Bloom’s taxonomy and enable them to think critically rather than memorise textbooks.”*

P11: *“Let’s not just use lectures alone…we need more strategies to teach our students and promote active learning and critical thinking…maybe we can try out something like problem-based learning or case studies.”*

## Discussion

The present phenomenology study highlighted the crucial role of dental materials science in dental education by exploring the experiences of dental educators in teaching dental materials science. Based on the present findings, dental educators have recognised its significance in clinical decision-making, patient outcomes, and the provision of evidence-based care in clinical practice. These findings were encouraging as educators themselves must embrace the importance of their subject taught in enabling their self-initiatives to integrate the subject content into the curricula, accomplish their duties and responsibilities well, and be motivated to improve their pedagogies [21]. Moreover, dental educators who appreciate their subjects and roles as educators might be able to promote the development of their professional identities [22].

The goal of dentistry is to improve patients’ quality of life by preventing oral diseases, reducing pain, and enhancing mastication, speech, and aesthetics. As many of these objectives would necessitate the placement of a diverse range of materials in the oral cavity, knowledge of dental materials science is critical for the selection of a biocompatible and long-lasting material that can withstand adverse conditions in the oral environment [23]. Dental materials science subjects allow dental students to gain a thorough understanding of the mechanical and physical properties of various dental materials, which is critical because it allows them to predict clinical performance and analyse the root cause of failure, allowing them to provide patients with optimal and evidence-based care [24]. Furthermore, participants felt that mastering dental material science should be a lifelong learning process, as newer and better materials are being introduced into the market at a staggering pace [11]. In addition, several techniques have been suggested to encourage life-long learning, including workplace learning, being in charge, seeking input from others, and using evidence at the point-of-care [25].

Limited instructional time, complexity of dental materials science as a subject matter, and insufficient resources were identified as challenges faced by dental educators in teaching dental materials science subjects. These challenges need to be addressed to optimise teaching and learning in dental materials science. First, limited classroom time coupled with overloaded curricula rendered teaching this subject challenging, as highlighted by the participants in the present study. The issue of limited instructional time is rooted in the curriculum design for identifying the learning needs of dental students. Instructional time is often proportionally allocated based on the importance of subjects. Similar to medical training which comprises a wide range of specialities, different specialities can argue on what dental students should learn depending on the impression that certain subjects are less important than others [26]. Second, the topics that need to be covered in dental materials science can be excessively broad, owing to their multidisciplinary nature [8]. Arguments also exist in the literature on whether it should be integrated into individual dental disciplines or taught as a stand-alone subject [6, 8]. While dental materials science is a complex subject and perceived to be difficult, innovative pedagogies are suggested to motivate students and help manage their cognitive load.

A systematic review of active learning in dental materials science recommended the implementation of flipped classrooms, clinical-based learning, computer-assisted learning, group discussion, microteaching with the BOPPPS (bridge-in, learning objective, pre-test, participatory learning, post-test, and summary) model, and game-based learning [12]. This is in line with a previous study revealed that the majority of students experienced difficulty in understanding concepts in dental material science with didactic lectures alone [7]. Third, insufficient resources are undeniably a common challenge for effective teaching [26, 27]. Dental education is expensive, and there is a compelling conflict of interest between profit- and non-profit-generating costs. Most participants acknowledged that they lacked sufficient resources to teach the subject, ranging from testing machines and laboratory facilities to manpower. However, the authors presumed that the impact of constraints on resources would lessen as virtual laboratories and interactive virtual simulators have become more common in recent years [28]. For instance, in a randomised controlled trial, dental students acquired better knowledge and skills for zinc phosphate cement manipulation by engaging in virtual learning objects [29].

The strategies suggested by dental educators provide valuable insight into effective teaching practices. Interactive teaching methods, clinical integration, and promoting critical thinking skills have emerged as important strategies to enhance teaching and learning experiences. While conventional lectures remain as the cornerstone of most dental educational systems, new teaching methodologies are required to encourage the less active students to be responsible for their educational agenda [30, 31]. Indeed, dental material science is often perceived as a dull subject because of its factual content involving the materials’ composition, properties, and the manner in which they interact with their surroundings [7]. Thus, an interactive teaching strategy along with the incorporation of clinical scenarios in the teaching of dental material science is required to enhance active learning and critical thinking [1]. This will also allow students to use their knowledge to address clinically-related situations [10].

Dental educators in the present study suggested more practical sessions for teaching dental materials science. The lack of practical sessions has made it challenging for students to relate their theoretical knowledge to their clinical significance. Hands-on practical laboratory sessions to select and manipulate various materials for different hypothetical clinical scenarios could enhance students’ learning experiences and prepare them for subsequent clinical years. This is consistent with experiential learning theory which emphasises the importance of acquiring knowledge and skills through engagement in real-world situations [32]. In addition, in responding to the need for training for innovative pedagogies in a limited resources context, needs assessment may be conducted to prioritise faculty development programmes. A needs assessment was conducted among Laos dental educators, who identified knowledge in developing syllabi and teaching using simulation as a priority for faculty development programmes in this resourceless country [27].

To the best of the authors’ knowledge, the present study is the first to explore dental educators’ perceptions in teaching dental materials science. The trustworthiness of the present qualitative study was enhanced by describing the background of the participants, triangulation of analysts, and member checking [33]. The variety of participants was a strength of the present study since it recruited participants with a range of teaching experiences from six different countries across East and Southeast Asian. It is worthwhile to study East and Southeast Asian dental education as they are different from the Western context. East and Southeast Asian students are often described as passive learners who prefer memorisation-based learning [34]. Dental schools in Asia continue to adopt traditional (discipline-based) curricula rather than embracing modern (integrated) curricula. Recognising these differences, many useful findings from past studies conducted in Western countries cannot be transferable to Asian dental schools.

However, the present study has some limitations. Since the study only interviewed thirteen participants, generalisation of the findings may be constrained. In addition, it is possible to assume that some distinct themes might emerge if future investigations are conducted among dental educators from other countries. Further research is warranted to investigate dental students’ perceptions and explore the effectiveness of specific teaching strategies in dental materials science. Meanwhile, longitudinal studies can assess the impact of improved teaching practices on students’ knowledge retention and clinical performance.

## Conclusion

Dental materials science is a crucial subject in the dental curriculum and plays a significant role in preparing dental students for clinical practice. This qualitative study explored the experiences of dental educators in teaching dental materials science and identified key themes related to the importance of the subject, the challenges faced in teaching it, and strategies to enhance teaching and learning. Understanding the perspectives of dental educators is essential for improving dental materials science education. The insights gained from the present study can inform future curriculum enhancements, teaching methodologies, and faculty training programmes. By addressing the identified challenges and implementing effective strategies, dental education can be enhanced, ultimately improving the knowledge and skills of dental students in the field of dental materials science.

### Electronic supplementary material

Below is the link to the electronic supplementary material.

Supplementary Material 1: The COREQ checklist for the present study

## Data Availability

All data generated or analysed during this study are included in this published article.

## Abbreviations

AIMST: Asian Institute of Medicine, Science and Technology University
COREQ: Consolidated Criteria for Reporting Qualitative Research
