# Supplementary material for: A phenomenological study on East and Southeast Asian dental educators: perceived importance, challenges, and strategies in teaching dental materials science
Source: BMC Oral Health. 2023 Aug 13;23:571. doi: 10.1186/s12903-023-03293-4 (PMC10424391; doi:10.1186/s12903-023-03293-4)
Supplement: Supplementary file 1 — Supplementary Material 1: The COREQ checklist for the present study [file 12903_2023_3293_MOESM1_ESM.pdf]

Consolidated criteria for reporting qualitative studies (COREQ): 32-item checklist

Developed from:
Tong A, Sainsbury P, Craig J. Consolidated criteria for reporting qualitative research (COREQ): a 32-item checklist for interviews and focus groups. *International Journal for Quality in Health Care*. 2007. Volume 19, Number 6: pp. 349 – 357

MANUSCRIPT TITLE:

No. Item	Guide questions/description	Reported on Page #
**Domain 1: Research team and reflexivity**		
Personal Characteristics		
1. Inter viewer/facilitator	Which author/s conducted the interview or focus group?	3
2. Credentials	What were the researcher's credentials? (E.g. PhD, MD)	3
3. Occupation	What was their occupation at the time of the study?	3
4. Gender	Was the researcher male or female?	3
5. Experience and training	What experience or training did the researcher have?	3
Relationship with participants		
6. Relationship established	*Was a relationship established prior to study commencement?* .	3
7. Participant knowledge of the interviewer	*What did the participants know about the researcher? (e.g. personal goals, reasons for doing the research).*	3
8. Interviewer characteristics	*What characteristics were reported about the interviewer/facilitator? (e.g. Bias, assumptions, reasons and interests in the research topic)*	3
**Domain 2: Study design**		
Theoretical framework		
9. Methodological orientation and Theory	*What methodological orientation was stated to underpin the study? (e.g. grounded theory, discourse analysis, ethnography, phenomenology, content analysis).*	3

Participant selection		
10. Sampling	*How were participants selected? (e.g. purposive, convenience, consecutive, snowball)*	3
11. Method of approach	*How were participants approached? (e.g. face-to-face, telephone, mail, email)*	3
12. Sample size	*How many participants were in the study?*	3
13. Non-participation	*How many people refused to participate or dropped out? Reasons?*	N/A
Setting		
14. Setting of data collection	*Where was the data collected? (e.g. home, clinic, workplace)*	3
15. Presence of non-participants	*Was anyone else present besides the participants and researchers?*	3
16. Description of sample	*What are the important characteristics of the sample? (e.g. demographic data, date)*	4
Data collection		
17. Interview guide	*Were questions, prompts, guides provided by the authors? Was it pilot tested?*	3
18. Repeat interviews	*Were repeat interviews carried out? If yes, how many?*	3
19. Audio/visual recording	*Did the research use audio or visual recording to collect the data?*	3
20. Field notes	*Were field notes made during and/or after the interview or focus group?*	N/A
21. Duration	*What was the duration of the inter views or focus group?*	3
22. Data saturation	Was data saturation discussed?	4
23. Transcripts returned	*Were transcripts returned to participants for comment and/or correction?*	3
**Domain 3: analysis and findings**		
Data analysis		
24. Number of data coders	How many data coders coded the data?	3

25. Description of the coding tree	*Did authors provide a description of the coding tree?*	N/A
26. Derivation of themes	*Were themes identified in advance or derived from the data?*	4
27. Software	*What software, if applicable, was used to manage the data?*	4
28. Participant checking	*Did participants provide feedback on the findings?*	3
Reporting		
29. Quotations presented	*Were participant quotations presented to illustrate the themes/findings? Was each quotation identified? (e.g. participant number)*	4-8
30. Data and findings consistent	*Was there consistency between the data presented and the findings?*	N/A
31. Clarity of major themes	*Were major themes clearly presented in the findings?*	4-8
32. Clarity of minor themes	Is there a description of diverse cases or discussion of minor themes?	4-8
